# Performance Evaluation of Cementless Composites with Alkali-Sulfate Activator for Field Application

**DOI:** 10.3390/ma13235410

**Published:** 2020-11-27

**Authors:** Jaehyun Lee, Taegyu Lee, Seungwoo Lee, Hyeonggil Choi

**Affiliations:** 1 Department of Safety Engineering, Seoul National University of Science and Technology, 232 Gongneung-ro, Nowon-gu, Seoul 01811, Korea; archi0528@seoultech.ac.kr; 2 Department of Fire and Disaster Prevention, Semyung University, 65 Semyung-ro, Jecheon-si, Chungbuk 27136, Korea; ltg777@semyung.ac.kr; 3 School of Architecture, Civil, Environment, and Energy Engineering, Kyungpook National University, Buk-Gu, Daegu 41566, Korea; woolee@knu.ac.kr

**Keywords:** alkali-activated composites (AAC), alkali-sulfate activator, engineering properties, acid resistance properties, CO_2_ reduction properties

## Abstract

This study analyzed the performance evaluation of alkali-activated composites (AAC) with an alkali-sulfate activator and determined the expected effects of applying AACs to actual sites. Results revealed that when the binder weight was increased by 100 kg/m^3^ at 7 days of age, the homogel strength of ordinary Portland cement (OPC) and AAC increased by 0.9 and 5.0 MPa, respectively. According to the analysis of the matrix microstructures at 7 days of age, calcium silicate hydrates (C–S–H, Ca_1.5_SiO_3.5_·H_2_O) and ettringite (Ca_6_Al_2_(SO_4_)_3_(OH)_12_·26H_2_O) were formed in AAC, which are similar hydration products as found in OPC. Furthermore, the acid resistance analysis showed that the mass change of AAC in HCl and H_2_SO_4_ solutions ranged from 36.1% to 88.0%, lower than that of OPC, indicating AAC’s superior acid resistance. Moreover, the OPC and AAC binder weight ranges satisfying the target geltime (20–50 s) were estimated as 180.1–471.1 kg/m^3^ and 261.2–469.9 kg/m^3^, respectively, and the global warming potential (GWP) according to binder weight range was 102.3–257.3 kg CO_2_ eq/m^3^ and 72.9–126.0 kg CO_2_ eq/m^3^. Therefore, by applying AAC to actual sites, GWP is expected to be 29.5 (28.8%)–131.3 (51.0%) kg CO_2_ eq/m^3^ less than that of OPC.

## 1. Introduction

Portland cement is the most widely used binder in the concrete industry. According to the Activity Report [1] published by the European Cement Association, global cement production in G20 countries, which are main world producers, is estimated to be about 4.1 billion tons in 2017. Among the different types of Portland cement, ordinary Portland cement (OPC)-based concrete is the most widely used [2], owing to its excellent performance and advantages as an economical construction material. To date, cement is irreplaceable as a building material [3].

However, Portland cement is reported to contribute 5–6% of the annual global CO_2_ emissions [2], which is relatively high, resulting in environmental issues worldwide. Accordingly, researchers are investigating alternatives to replace cement [3,4]. Although completely replacing cement in the construction industry is challenging, it can be partially replaced with binders that are suitable for different structural purposes. Thus, numerous researchers have studied a variety of materials for cement replacement [5,6,7,8].

Among them, ground granulated blast-furnace slag (GGBS) and fly ash (FA) can exhibit the same performance as the OPC. When GGBS and FA are used to partially replace cement, they are called an alkali-activated composite (AAC). AAC is garnering attention as an environmentally friendly material that can enhance the performance of concrete through its high initial strength and excellent durability. Accordingly, many researchers have investigated the performance of materials using AAC [9,10]. Cartwright et al. [11] found that AAC exhibited 3–6 times more shrinkage than OPC. Likewise, Kumarappa et al. [12] and Atis et al. [13] reported that AAC showed considerably higher shrinkage than OPC, which can lead to serious issues when used in structures. In response, Choi et al. [14] added a calcium sulfoaluminate-based expansive additive (CSA; 0%, 2.5%, 5.0%, and 7.5% based on the mass of the binder) to compensate for the shrinkage of AAC mortar. Researchers reported that, when the elastic modulus was low, CSA greatly compensated for the dry shrinkage of AAC mortar in the initial curing period. Therefore, research on suitable mixing materials to compensate for shrinkage is important for applying AAC.

In developing new resources and reusing waste for sustainable development, circulating fluidized bed combustion (CFBC) ash has recently become one of the most widely used combustion technologies [15,16]. CFBC, a clean coal technology, is commonly used as an eco-friendly power generation solution due to its effective control of sulfur oxide and nitrogen oxide emissions from coal in thermal power plants [17,18]. To promote the sustainable development of CFBC power generation technology, much attention has been paid to recycling based on the self-hardening properties of CFBC ash, a fundamental physical and chemical property. Specifically, in countries operating large-scale CFBC power plants, such as China and Korea, researchers are investigating the use of CFBC ash as a binder for low-strength materials [19]. Such studies include research on whether a combination of CFBC and GGBS can be used as a binder in the compression production of bricks, research on technology for enhancing the bonding strength of the cement mixture by adding CFA and additives to the aluminate cement of lightweight brick, and research on the use of CFBC ash as a binder for stabilizing soil [16,20,21]. Furthermore, CFBC is characterized by its superior performance and few pollutant emissions compared to conventional pulverized coal combustion technology. Moreover, researchers have reported its application to petroleum coke, which is suitable for a variety of fuels and has sulfur content of less than 5%, follows the principle of waste recycling [22].

However, CFBC has considerable disadvantages that have impeded its adoption in the industry. For example, using a large amount of FA in OPC-based concrete increases the setting time and degrades the initial mechanical properties. Moreover, for the alkaline activation of FA, an appropriate alkaline silicate and/or hydroxide containing a high alkali activator must be used [23,24,25]. These problems limit the sustainable benefits afforded by the CFBC system. Consequently, researchers have attempted to alleviate some of the concerns regarding alkali activation systems using powder activators, with some success [26,27]. Studies have investigated the use of neutral salts, such as sodium sulfate and sodium carbonate, as FA activators in large amounts of FA-based concrete as an alternative to sustainable concrete [28,29,30].

**Figure 1:**
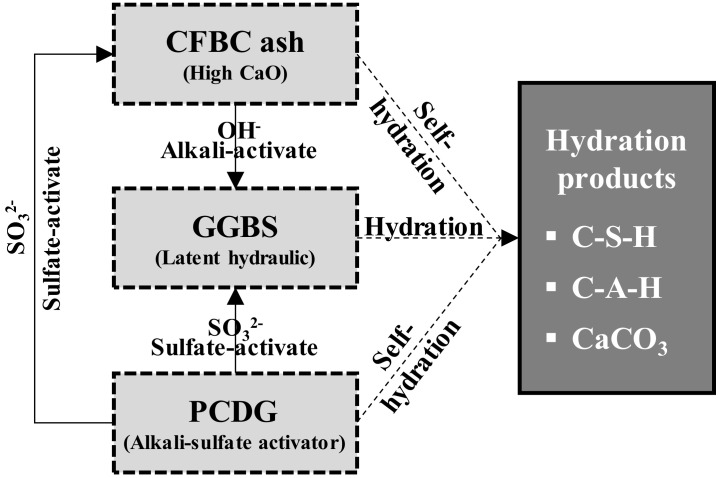
Chemical reaction mechanism diagram of alkali-activated composites (AAC).

However, there is no research on AAC as a replacement for OPC that can be applied to actual sites. Moreover, no studies have proposed an optimal mixing guide using local AAC materials or presented the expected effects of AAC through calculations of its impact on the environment. Accordingly, this study derived the composition of AAC with alkali-sulfate activator that can replace OPC based on previous research. The chemical reaction mechanism [31] for this material is shown in Figure 1.

This study aims to analyze the engineering properties, acid resistance, and CO_2_ reduction properties of alkali-activated composites (AAC) with an alkali-sulfate activator and determine the expected effects of applying AAC to actual sites. Experiments were conducted with liquid B, where the water/binder ratio was set to 100, 120, and 140%, and the replacement ratio to 50%, 60%, and 70%. In series I, raw material analysis was conducted via scanning electron microscopy and X-ray fluorescence. In series II, for the engineering properties analysis, the geltime and homogel strength were measured, after which the microstructure of the matrix was analyzed using a scanning electron microscope. Subsequently, in series III, the acid resistance and CO_2_ reduction properties were evaluated through mass change and global warming potential (GWP) analyses.

## 2. Experimental Procedure

### 2.1. Materials

**Figure 2:**
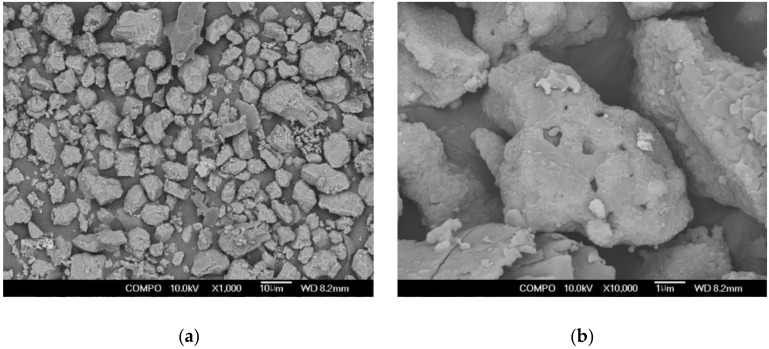
Scanning electron microscope of petro cokes desulfurization gypsum (PCDG) at (**a**) 1000× and (**b**) 10,000× magnification.

**Figure 3:**
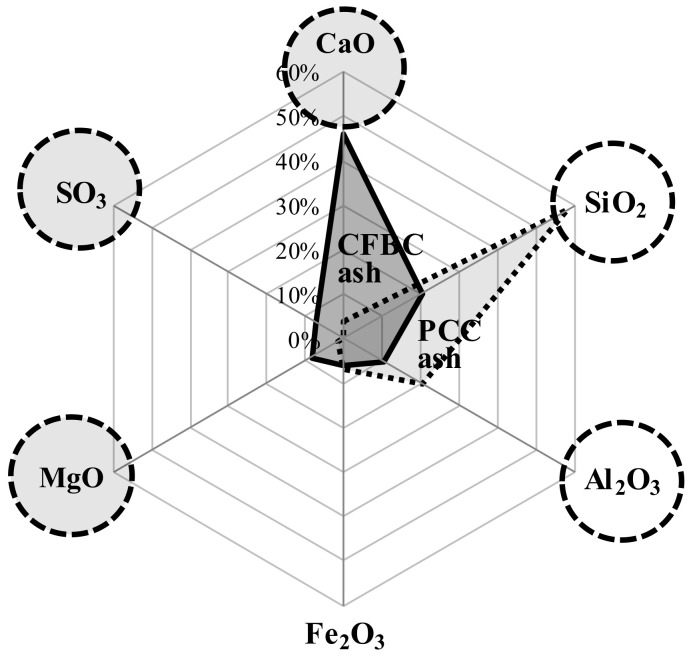
Chemical compositions of circulating fluidized bed combustion (CFBC) ash and pulverized coal combustion (PCC) ash.

Table 1 shows the chemical compositions of OPC (Halla Cement, Gangneung-si, Korea), AAC (Zian Corporation, Wanju-gun, Korea), and sodium silicate solution (SSS, Youngil Chemical, Incheon, Korea) used in this study. AAC had higher content of SiO_2_, Al_2_O_3_, and SO_3_ than OPC. SSS was composed of SiO_2_, Fe_2_O_3_, H_2_O, and Na_2_O. Table 2 shows the physical properties of the materials used. For OPC, Type 1 (KS L 5201) with a specific surface area of 3120 cm^2^/g was used [32], and for AAC, a mixture of GGBS, CFBC ash, and petro cokes desulfurization gypsum (PCDG) with a specific surface area of 4120 cm^2^/g was used. In particular, PCDG acts as an alkali-sulfate activator with a granular geometry as shown by the SEM analysis in Figure 2. From Figure 3, the chemical composition of CFBC ash differs from the existing pulverized coal combustion (PCC) ash. CFBC ash has a higher content of CaO, MgO, and SO_3_ than PCC ash. Meanwhile, for SSS, Type 3 (KS M 1415) with water insolubility of 0.0026 was used [33].

### 2.2. Experimental Procedures

Table 3 shows the experimental plan of this study. The binder type of liquid B was set to two levels (OPC and AAC), the W/B ratio to three levels (100, 120, and 140%), and the replacement ratio to three levels (50, 60, and 70%). In series I, measurements were conducted for the raw material analysis via scanning electron microscopy (Emcrafts, Gwangju-si, Korea) and X-ray fluorescence (Malvern Panalytical, Seongnam-si, Korea). In series II, for the engineering properties analysis, the geltime and homogel strength were measured and SEM analysis was performed. Then, in series III, the mass change and global warming potential (GWP) were evaluated for the acid resistance and CO_2_ reduction properties analysis. Table 4 displays the mixing proportions used.

### 2.3. Test Methods

Table 5 shows the test method for each evaluation item. Tests were performed for each evaluation item (scanning electron microscope, X-ray fluorescence) in series I according to American Society for Testing and Materials (ASTM) C1723 [34] and ASTM C114 [35], respectively. For series II, the geltime test was conducted according to ASTM D4217 [36], the homogel strength to ASTM C109 [37], and SEM to ASTM C1723 [34]. The target geltime was set to 20–50 s, and the target homogel strength to at least 2 MPa [10].

For Series III, mass change and GWP were evaluated in accordance with the test methods ASTM C267 [38], 579 [39], and International Organization for Standardization (ISO) 14040 [40]. In particular, the characterized environmental impacts index for evaluating GWP was calculated based on the LCI DB [41,42]. The index is shown in Table 6. In particular, simple regression analysis was used to analyze the statistical correlation of each variable obtained as a test result.

## 3. Results and Discussion

### 3.1. Engineering Properties Analysis

**Figure 4:**
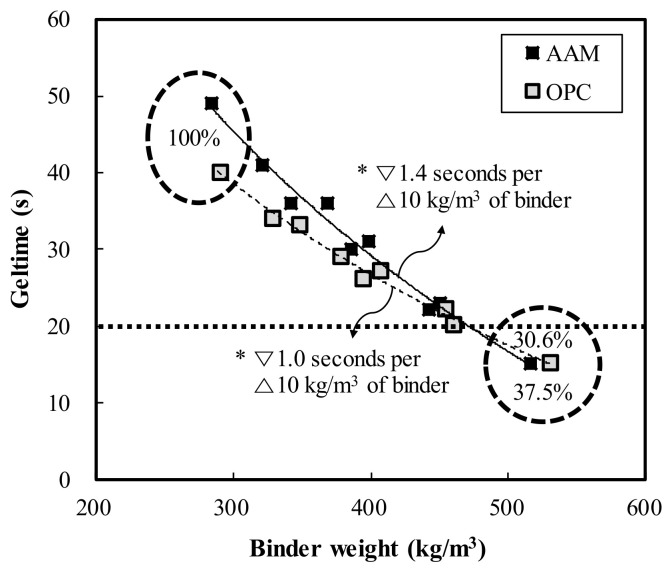
Geltime of OPC and AAC as a function of binder weight.

Figure 4 shows the change in geltime according to the binder weight of OPC and AAC. In both OPC and AAC, the geltime tended to decrease as the binder weight increased. In OPC, when the binder weight increased from 291.1 kg/m^3^ to 531.3 kg/m^3^, the geltime decreased from 40 s (100%) to 15 s (37.5%). When the binder weight increased by increments of 10 kg/m^3^, the geltime decreased by 1.0 s per increment. Meanwhile, in AAC, when the binder weight increased from 285.0 kg/m^3^ to 516.8 kg/m^3^, the geltime decreased from 49 s (100%) to 15 s (30.6%). When the binder weight increased by increments of 10 kg/m^3^, the geltime decreased by 1.4 s per increment. Under similar mixing conditions, the OPC test specimen showed lower geltime than AAC overall, demonstrating that OPC is more advantageous for shortening the geltime than AAC.

**Figure 5:**
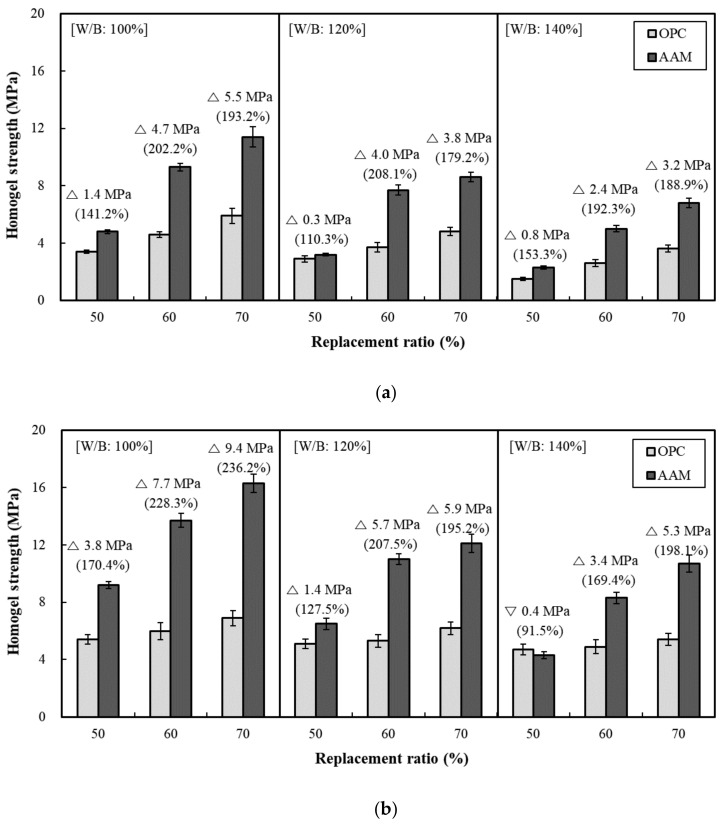
Homogel strength of OPC and AAC by replacement ratio: (**a**) 3 days (**b**) 7 days.

Figure 5 illustrates the homogel strength according to the replacement ratio of OPC and AAC. Figure 5a shows the results at 3 days of age; the test specimen exhibited the highest homogel strength at a replacement ratio of 70%. At W/B ratios of 100%, 120%, and 140%, and a replacement ratio of 70%, AAC exhibited higher homogel strength than OPC by 5.5 MPa (193.2%), 3.8 MPa (179.2%), and 3.2 MPa (188.9%), respectively. Meanwhile, Figure 5b shows the homogel strength according to W/B ratios of 100%, 120%, and 140% at a replacement ratio of 70% at 7 days of age, at which AAC exhibited higher homogel strength than OPC by 9.4 MPa (236.2%), 5.9 MPa (195.2%), and 5.3 MPa (198.1%), respectively. As the age increased from 3 days to 7 days, the difference in homogel strength between most AAC specimens and OPC increased. Furthermore, in both OPC and AAC, the homogel strength tended to increase when W/B decreased and the replacement ratio increased. Under the same mixing conditions, the AAC specimens exhibited homogel strength up to 236.2% higher than OPC, demonstrating that AAC is more suitable for securing homogel strength than OPC.

**Figure 6:**
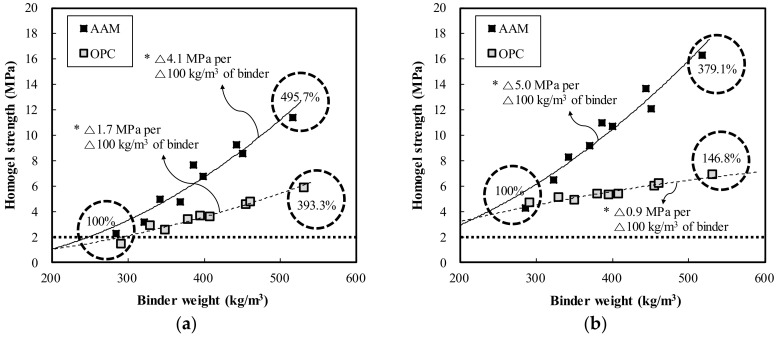
Homogel strength of OPC and AAC by binder weight: (**a**) 3 days (**b**) 7 days.

**Figure 7:**
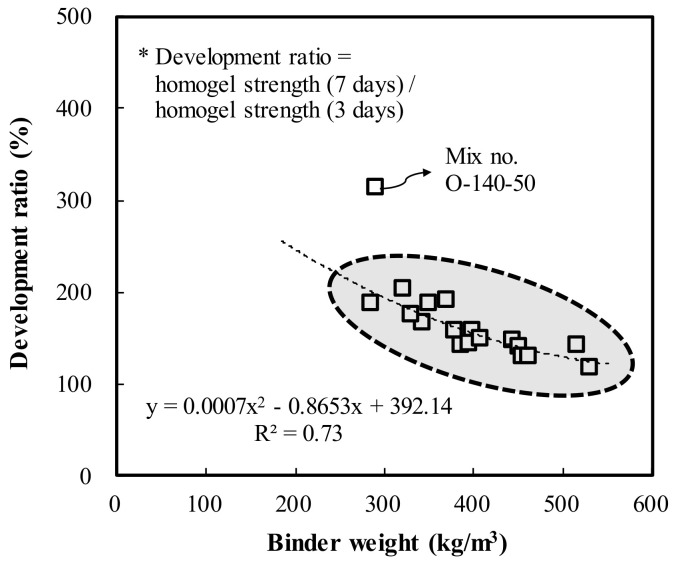
Development ratio of OPC and AAC by binder weight.

Figure 6 shows the homogel strength according to increases in binder weight of the OPC and AAC specimens. The homogel strength tended to increase as the binder weight increased. At 3 days of age, OPC showed an increase of 1.7 MPa when the binder weight increased by 100 kg/m^3^, whereas AAC showed an increase of 4.1 MPa. This is illustrated in Figure 6a. The ratios of maximum homogel strength to minimum homogel strength of OPC and AAC were 393.3% and 495.7%, respectively. Figure 6b displays the results at 7 days of age, at which OPC showed an increase of 0.9 MPa when the binder weight increased by 100 kg/m^3^, whereas AAC showed an increase of 5.0 MPa. The ratios of maximum homogel strength and minimum homogel strength of OPC and AAC were 146.8% and 379.1%, respectively. These results indicate that the strength enhancement effect according to binder weight of the AAC specimen was greater than that of OPC. For further analysis, the relationship between the increase rate of strength by age according to the increases in binder weight was expressed as a regression line, as shown in Figure 7. According to the results, as the binder weight increases, the increase rate decreases, and the equation y = 0.0007x^2^ − 0.8653x + 392.14 was derived for all specimens excluding Mix number O-140-50.

**Figure 8:**
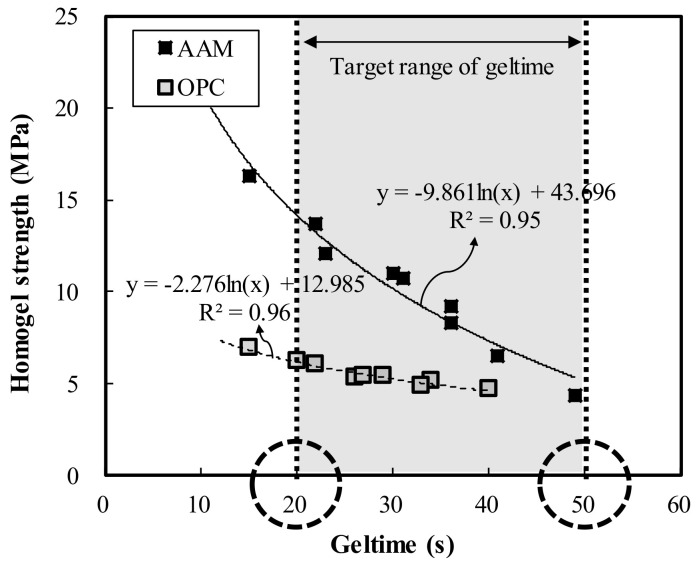
Correlations between geltime and homogel strength.

**Figure 9:**
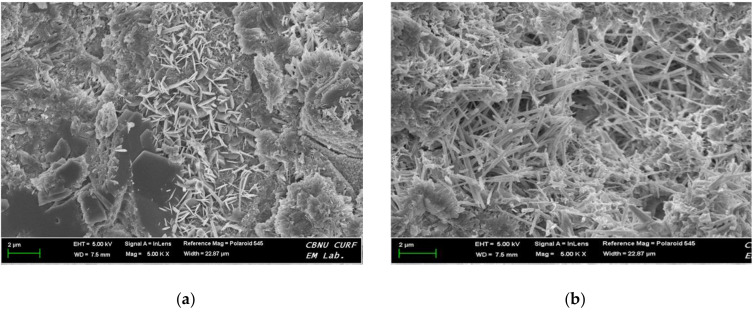
Scanning electron microscope images: (**a**) OPC, (**b**) AAC at 7 days (5000× magnification).

Figure 8 shows the relationship between geltime and homogel strength, which was found to be inversely related. The regression equations derived for OPC and AAC were y = −2.276ln(x) + 12.985 and y = −9.861ln(x) + 43.696, respectively. SEM images at 5000× magnification were taken to analyze the microstructure of the hardened OPC and AAC specimens at 7 days of age, as shown in Figure 9. From the results, similar hydration products as in OPC, calcium silicate hydrates (C–S–H, Ca_1.5_SiO_3.5_·H_2_O)) and ettringite (Ca_6_Al_2_(SO_4_)_3_(OH)_12_·26H_2_O), were formed in AAC. These findings proved that in the AAC specimens, GGBS, CFBC ash, and PCDG produced hydrates similar to OPC through chemical hydration reactions even though OPC was not used.

### 3.2. Acid Resistance Analysis

**Figure 10:**
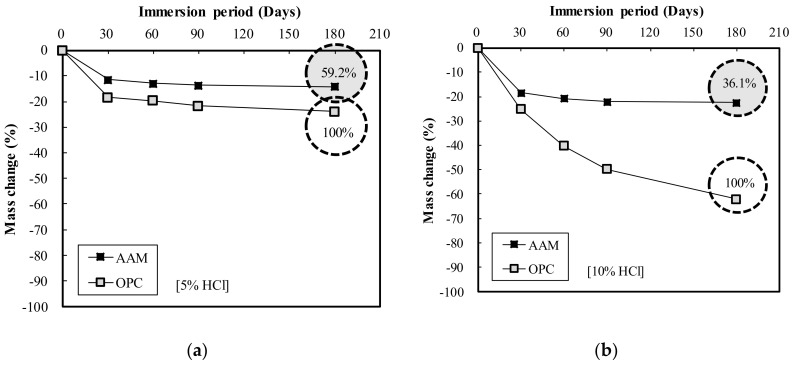
Mass change of OPC and AAC by immersion period in (**a**) 5% HCl and (**b**) 10% HCl solution.

Figure 10 shows the mass change measurements according to the immersion period of the OPC and AAC specimens in HCl solution. Figure 10a illustrates the results of immersion in 5% HCl solution, in which both OPC and AAC exhibited a sharp decrease in mass change until 30 days of immersion, after which they showed a more gradual decrease. After 180 days of immersion, the mass change of AAC was low at 59.2% of OPC. When immersed in 10% HCI solution as illustrated in Figure 10b, AAC exhibited a sharp decrease in mass change until 30 days of immersion, after which mass change gradually decreased, while OPC showed a rapid decrease throughout the immersion period up to 180 days. At 180 days of immersion, the mass change of AAC was low at 36.1% of that for OPC.

**Figure 11:**
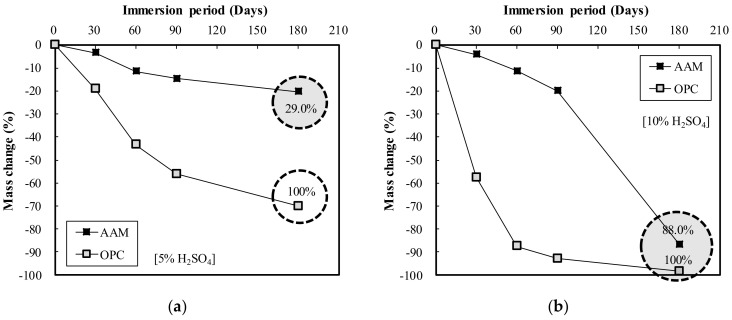
Mass change of OPC and AAC by immersion period: (**a**) 5% H_2_SO_4_ (**b**) 10% H_2_SO_4_ solution.

Meanwhile, Figure 11 displays the mass change according to the immersion period of the OPC and AAC specimens in H_2_SO_4_ solution. In Figure 11a, the results of immersion in 5% H_2_SO_4_ solution indicate that mass change decreased rapidly for OPC and gradually for AAC. After 180 days of immersion, the mass change of AAC was low at 29.0% of that for OPC. Conversely, in 10% H_2_SO_4_ solution as shown in Figure 11b, the mass change of AAC was somewhat lower at 88.0% of that for OPC. The mass change of AAC gradually decreased until 90 days of immersion, after which it rapidly decreased; in contrast, for OPC, mass change rapidly decreased in 60 days of immersion and then gradually decreased.

The acid resistance properties analysis showed that the mass change of AAC in HCl and H_2_SO_4_ solutions ranged from 36.1 to 88.0%, lower than that of OPC, indicating AAC’s superior acid resistance compared to OPC. From the explanation in “3.1. Engineering Properties Analysis,” the homogel strength of the AAC specimen is higher than that of OPC under the same mixing conditions. Therefore, its acid resistance is related to its excellent properties.

### 3.3. CO_2_ Reduction Properties Analysis

**Figure 12:**
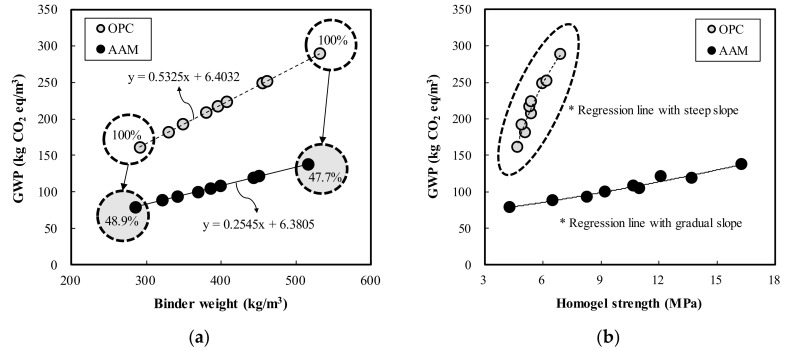
Global warming potential (GWP) of OPC and AAC: (**a**) binder weight, (**b**) compressive strength.

Figure 12 shows a comparative analysis of GWP of the OPC and AAC specimens. According to the analysis of change in GWP relative to binder weight in Figure 12a, GWP tended to increase as the binder weight increased. For OPC, GWP increased by 25.45 kg CO_2_ eq with a 100 kg/m^3^ increase in binder weight, while GWP of AAC increased by 53.25 kg CO_2_ eq. Thus, under the same mixing conditions, the GWP of AAC was low at 47.7 to 48.9% of OPC. Figure 12b illustrates the change in GWP according to the homogel strength; both OPC and AAC exhibited an increase in GWP as the homogel strength increased. In particular, for OPC, the relationship between homogel strength and GWP presented a steeper slope than that of AAC. This demonstrates that AAC has a greater effect on CO_2_ reduction than OPC.

**Figure 13:**
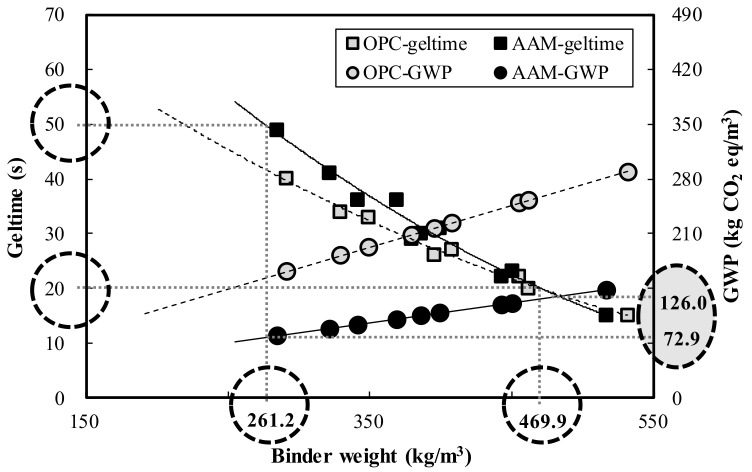
OPC and AAC binder weight range and GWP that satisfy the target geltime.

Figure 13 shows the binder weight range and GWP of OPC and AAC that satisfy the target geltime range (20–50 s). For reference, all specimens satisfying the target geltime range exceeded the target homogel strength of 2 MPa. According to the analysis, the OPC and AAC binder weight ranges satisfying the target geltime range were estimated at 180.1–471.1 kg/m^3^ and 261.2–469.9 kg/m^3^, respectively. Moreover, GWP in relation to binder, weight range was 102.3–257.3 kg of CO_2_ eq/m^3^ and 72.9–126.0 kg of CO_2_ eq/m^3^. Therefore, by applying AAC to actual sites under conditions that satisfy the target geltime range, the GWP was expected to be 29.5 (28.8%)–131.3 (51.0%) kg CO_2_ eq/m^3^ less than that of OPC.

## 4. Conclusions

This study analyzed the performance evaluation of alkali-activated composites (AAC) with an alkali-sulfate activator. The results are summarized as follows:

1. Geltime decreased by 1.0 and 1.4 s in OPC and AAC, respectively, for each increase in binder weight of 10 kg/m^3^. Thus, under similar mixing conditions, OPC had more advantageous properties with a shorter geltime than AAC.
2. In both OPC and AAC specimens, the homogel strength tended to increase as W/B decreased and the replacement ratio increased. At 7 days of age, W/B ratios of 100%, 120%, and 140%, and a replacement ratio of 70%, the AAC specimens exhibited higher homogel strength by 9.4 MPa (236.2%), 5.9 MPa (195.2%), and 5.3 MPa (198.1%), respectively. Hence, under the same mixing conditions, AAC is more advantageous for securing homogel strength than OPC.
3. At 3 days of age, OPC showed an increase of 1.7 MPa when the binder weight increased by 100 kg/m^3^, whereas AAC showed an increase of 4.1 MPa. In addition, at 7 days of age, OPC showed an increase of 0.9 MPa when the binder weight increased by 100 kg/m^3^, whereas AAC showed an increase of 5.0 MPa. Geltime and homogel strength were found to be inversely related for both OPC and AAC.
4. Based on the 5000× magnification SEM images taken at 7 days to analyze the matrix microstructures, similar hydration products found in OPC, calcium silicate hydrates (C–S–H, Ca_1.5_SiO_3.5_·H_2_O)) and ettringite (Ca_6_Al_2_(SO_4_)_3_(OH)_12_·26H_2_O), were formed in AAC. These findings proved that in the AAC specimens, GGBS, CFBC ash, and PCDG produced hydrates similar to OPC through chemical hydration reactions, although OPC was not used.
5. The acid resistance properties analysis showed that the mass change of AAC in HCl and H_2_SO_4_ solutions ranged from 36.1 to 88.0%, lower than that of OPC, indicating AAC’s superior acid resistance. Furthermore, for OPC, GWP increased by 25.45 kg CO_2_ eq with a 100 kg/m^3^ increase in binder weight, while GWP of AAC increased by 53.25 kg CO_2_ eq. This demonstrates that AAC has a greater effect on CO_2_ reduction than OPC.

The OPC and AAC binder weight ranges satisfying the target geltime (20–50 s) were estimated at 180.1–471.1 kg/m^3^ and 261.2–469.9 kg/m^3^, respectively, and the GWP according to binder weight range was 102.3–257.3 kg CO_2_ eq/m^3^ and 72.9–126.0 kg CO_2_ eq/m^3^, respectively. Therefore, by applying AAC to actual sites, GWP is expected to be 29.5 (28.8%)–131.3 (51.0%) kg CO_2_ eq/m^3^ less than that of OPC.

In this study, raw material analysis, engineering properties analysis, acid resistance and CO_2_ reduction properties analysis were performed as basic research on AAC. In the future, in-depth tests of various mechanical properties (tensile stress, shear stress, elastic deformation, modulus of elasticity, etc.) for AAC will be conducted. In addition, a detailed evaluation of the life cycle assessment and environmental impact assessment (global warming potential, ozone layer depletion potential, acidification potential, eutrophication potential, photochemical ozone creation potential, abiotic depletion potential, etc.) will be performed to analyze eco-friendly properties.

## Figures and Tables

**Table 1:** Chemical compositions of the used materials.

Material	Chemical Composition (%)								
	CaO	SiO_2_	Al_2_O_3_	Fe_2_O_3_	MgO	SO_3_	H_2_O	Na_2_O	Other
OPC ^(1)^	66.70	17.20	4.38	3.13	3.03	3.48			2.08
AAC ^(2)^	56.28	20.72	8.29	0.51	2.31	10.51			1.38
SSS ^(3)^		27.2		0.01			63.6	9.14	0.05

^(1)^ OPC: Ordinary Portland cement; ^(2)^ AAC: Alkali-activated composites; ^(3)^ SSS: Sodium silicate solution.

**Table 2:** Physical properties of the materials.

Material	Property
OPC	Type 1 ordinary Portland cement (KS L 5201)
	Density: 3.15 g/cm^3^, specific surface area: 3120 cm^2^/g
AAC	Alkali-activated composites
	Density: 2.82 g/cm^3^, specific surface area: 4120 cm^2^/g
SSS	Type 3 Sodium silicate solution (KS M 1415)
	Density: 1.38 g/cm^3^, Water insolubility: 0.0026

**Table 3:** Experimental plan.

Series	Experimental Factor and Level			Evaluation Item
	Binder Type	W/B Ratio (%)	Replacement Ratio (%)	
Ⅰ. Raw material analysis	OPC AAC	100 120 140	50 60 70	Scanning electron microscope
				X-ray fluorescence
Ⅱ. Engineering properties analysis				Geltime (s)
				Homogel strength (MPa)
				Scanning electron microscope
Ⅲ. Acid resistance and CO_2_ reduction properties analysis				Mass change (%)
				Global warming potential (kg CO_2_ eq/m^3^)

**Table 4:** Mixing proportions.

Mix No.	Liquid A (kg/m^3^)		Liquid B (kg/m^3^)		Mix No.	Liquid A (kg/m^3^)		Liquid B (kg/m^3^)	
	SSS	Water	OPC	SSS		SSS	Water	AAC	Water
O-100-50	346.0	250.0	379.5	379.5	A-100-50	346.0	250.0	369.1	369.1
O-100-60	276.8	200.0	455.4	455.4	A-100-60	276.8	200.0	442.9	442.9
O-100-70	207.6	150.0	531.3	531.3	A-100-70	207.6	150.0	516.8	516.8
O-120-50	346.0	250.0	329.5	395.4	A-120-50	346.0	250.0	321.6	385.9
O-120-60	276.8	200.0	395.4	474.5	A-120-60	276.8	200.0	385.9	463.1
O-120-70	207.6	150.0	461.3	553.6	A-120-70	207.6	150.0	450.3	540.3
O-140-50	346.0	250.0	291.1	407.6	A-140-50	346.0	250.0	285.0	398.9
O-140-60	276.8	200.0	349.4	489.1	A-140-60	276.8	200.0	342.0	478.7
O-140-70	207.6	150.0	407.6	570.6	A-140-70	207.6	150.0	398.9	558.5

**Table 5:** Test methods by evaluation items.

Series	Evaluation Item	Test Method
Ⅰ. Raw material analysis	Scanning electron microscope	ASTM C1723
	X-ray fluorescence	ASTM C114
Ⅱ. Engineering properties analysis	Geltime (s)	ASTM D4217
	Homogel strength (MPa)	ASTM C109
	Scanning electron microscope	ASTM C1723
Ⅲ. Acid resistance and CO_2_ reduction properties analysis	Mass change (%)	ASTM C267, 579
	Global warming potential (kg CO_2_ eq/m^3^)	ISO 14040

**Table 6:** Characterized environmental impacts index of materials used.

Materials	GWP (kg CO_2_ eq.)
OPC	5.32 × 10^−1^
GGBS	5.01 × 10^−1^
CFBC ash	1.01 × 10^−2^
PCDG	5.96 × 10^−4^
SSS	1.73 × 10^−3^
Water	8.88 × 10^−3^

## Abbreviations

AAC	Alkali-activated Composites
OPC	Ordinary Portland Cement
GWP	Global Warming Potential
GGBS	Ground Granulated Blast-furnace Slag
FA	Fly Ash
CSA	Calcium Sulfoaluminate
CFBC	Circulating Fluidized Bed Combustion
PCDG	Petro Cokes Desulfurization Gypsum
PCC	Pulverized Coal Combustion
SSS	Sodium Silicate Solution
SEM	Scanning Electron Microscope
